# Treg/Th17在慢性阻塞性肺疾病合并肺癌中的相关研究进展

**DOI:** 10.3779/j.issn.1009-3419.2019.12.10

**Published:** 2019-12-20

**Authors:** 金花 周, 伟 王, 瑞娟 刘

**Affiliations:** 1 272000 济宁，济宁市第一人民医院呼吸内科 Jining No.1 People's Hospital, Jining 272000, China; 2 250033 济南，山东大学第二医院呼吸内科 The Second Hospital of Shandong University, Jinan, 250033, China

**Keywords:** 慢性阻塞性肺疾病, 肺肿瘤, Th17, Treg, 免疫, 肿瘤微环境, Chronic obstructive pulmonary disease, Lung neoplasms, Th17, Treg, immunization, Tumor microenvironment

## Abstract

慢性阻塞性肺疾病（chronic obstructive pulmonary disease, COPD）和肺癌为全球性高发病率、高死亡率疾病，严重增加社会经济负担。烟雾暴露、遗传易感性和慢性炎症等是二者发病的共同易感因素。目前异常炎症免疫反应在两种疾病的发生、发展中均有作用，在免疫应答过程中逐渐产生有利于血管生成和免疫抑制的肿瘤微环境（tumor microenvironment, TME），最终使肿瘤细胞发生免疫逃逸，导致肿瘤形成。本文就COPD合并肺癌的现状以及异常免疫应答尤其是Treg/Th17与其发生、发展关系方面做一个简要综述。

## Abstract (en)

Chronic obstructive pulmonary disease (COPD) and lung cancer are global high incidence and high mortality diseases, which seriously increase the socio-economic burden. Smoke exposure, genetic susceptibility and chronic inflammation are common susceptible factors. At present, abnormal inflammatory immune response plays an important role in the occurrence and development of the two diseases. In the process of immune response, tumor microenvironment (TME) is gradually produced, which is beneficial to angiogenesis and immunosuppression, and finally leads to immune escape of tumor cells, leading to tumor formation. In this paper, the present situation of COPD complicated with lung cancer and the relationship between abnormal immune response, especially Treg/Th17, and its occurrence and development are briefly reviewed.

## 1 前言

慢性阻塞性肺疾病（chronic obstructive pulmonary disease, COPD）目前被视为独立于吸烟之外可以导致肺癌的另一项危险因素^[1]^，二者发病率、死亡率逐年增高，相关的发病机制非常复杂，目前尚未定论，且在不同机制之间错综复杂、互为因果。近期研究^[2]^显示，COPD的存在影响肺癌的免疫微环境，且免疫治疗在COPD合并肺癌患者治疗中获益，提示COPD微环境中的免疫状态对肺癌的形成、进展及预后有影响。CD4^+^ T细胞是体内重要的免疫细胞，Th17和Treg细胞是其中主要的两种成分，Th17/Treg在调节炎症免疫及免疫耐受方面非常重要，尤其在慢性炎症继发肿瘤时炎癌转换过程中作用更为明显。针对COPD合并肺癌这一群体，存在长期慢性炎症诱导肺癌高发风险，Th17/Treg在两种疾病并存时的作用尚有争议。本文就Th17和Treg细胞在COPD合并肺癌发病中的作用及相关性研究进行概述。

## 2 COPD与肺癌

COPD与肺癌并存的现象严重，一项以肺癌病因学为基础的病例对照研究的环境和遗传学研究^[3]^表明，患有肺气肿和COPD的个体肺癌发病率增高，而患有COPD的吸烟者更容易合并肺癌。一项前瞻性研究^[4]^中显示，COPD合并肺癌中，以非小细胞肺癌（non-small cell lung cancer, NSCLC）多发，最常见的组织学类型是鳞状细胞癌，其次是腺癌和小细胞肺癌，而腺癌在COPD GOLD Ⅰ期中占主导地位，而鳞状细胞癌在COPD GOLD Ⅱ期和Ⅲ期中最为常见，COPD不但增加肺癌风险，且肺癌的发生率与COPD、肺功能的严重程度呈反比关系。但亦有研究^[5]^得出相反结论，即COPD肺功能越差，更容易合并肺癌。总之，COPD已成为肺癌发病的独立危险因素，并且肺癌也是COPD患者中最常见的死亡原因之一^[1]^。虽然肺癌的治疗手段越来越多，但当肺癌与COPD并存时，此类患者由于术中风险大，术后并发症多，化、放疗耐受性差，效果不理想，基因突变率低等，从临床观察，相对非单纯肺癌患者，上述治疗方案均获益相对较小，且表明COPD的存在影响吸烟者及Ⅳ期NSCLC患者的生存情况^[6-8]^。随着近年来免疫治疗在各种疾病中的治疗获益，免疫异常在疾病的发病过程中备受关注，肿瘤浸润免疫细胞和免疫检查点对肿瘤的发生、进展和生存预后均发挥着重要的作用^[9]^。进一步明确肺癌合并COPD时的免疫状态，将更有助于为这一疾病群体选择或制定更个体化的治疗方案。

## 3 经典辅助T细胞亚型在COPD与肺癌中的作用

在COPD患者气道和肺泡腔内，CD8细胞毒性T细胞是主要的T细胞，其数量随着气流受限和肺气肿分期的增加而明显增加，并且CD4^+^ T细胞的数量也是增加的^[10]^，在遇到特异性抗原时，初始CD4^+^ T细胞活化分化成为两种效应T细胞亚型发挥作用。Th1细胞是吞噬细胞介导的宿主免疫的主要效应细胞，其可分泌白介素2（interleukin 2, IL-2）、干扰素-γ（interferon γ, INF-γ）和肿瘤坏死因子α（tumor necrosis factor α, TNF-α），主要参与抵御细胞内病原体。而Th2细胞可分泌IL-4、IL-5、IL-6、IL-10等因子，参与变态反应和防御寄生虫感染。COPD和肺癌中的炎症细胞分布类似，但COPD免疫细胞与肺癌却不同。COPD中淋巴细胞类型主要为Th1细胞，巨噬细胞类型为M1、M2混合型，肺癌微环境主要是Th2淋巴细胞和M2型巨噬细胞^[11]^。既往研究^[12]^发现，Th1细胞分泌早幼粒细胞因子，具有抗肿瘤作用，Th2细胞分泌抗炎细胞因子，具有促进肿瘤作用，且Th1/Th2比值与肿瘤分期有关。研究^[13]^发现，COPD、AECOPD与健康者相比，Th1和Th2细胞均有增多，而Th1/Th2比值变化与AECOPD的严重程度及预后相关。研究^[14, 15]^表明，Th1比例增高是肺癌预后不良的一个指标，而另有研究表明Th1细胞比例增高可增强抗肿瘤免疫反应，高水平的Th1可以预测更好的化疗临床结果，而Th2细胞比例增高则下调抗肿瘤免疫反应，预测更差的化疗效果。Th17与Treg同属于T细胞亚群，与Th1和Th2之间的平衡相似的是，Th17和Treg细胞之间也存在一个重要的平衡，它们在维持免疫环境中扮演着重要角色，失衡会导致局部或全身的异常免疫反应，近年来，Th17和Treg细胞逐渐取代了Th1/Th2模式，常用于研究T细胞调控在炎症、肿瘤及自身免疫性疾病中作用^[16]^，Th17、Treg亦参与CODP、肺癌的发病机制。

## 4 Treg细胞在COPD合并肺癌中的作用

Treg细胞是T细胞亚群中的一种，通过抑制效应性T细胞的增殖、激活及B细胞产生免疫球蛋白等机制，在细胞分化和发挥免疫抑制的生物学功能中起重要的作用，特异性叉头状转录因子3（fork-head transcription factor p3, Foxp3）是其特异性转录因子。Treg细胞与患者的免疫功能有关，可间接反映肺部和气道的炎症程度，因此Treg细胞可能参与COPD的发病及病情变化过程。AECOPD患者在有强烈炎症信号的情况下表现出Tregs的代偿性增强，并与吸烟指数显著相关，有研究^[17]^证实严重的COPD与血液和支气管黏膜中较低水平的Tregs有关，而没有COPD的吸烟者中较高的Tregs水平表明Tregs对COPD的潜在保护作用。此外，即使在同一COPD患者中，Tregs细胞随着疾病的分期和采样点的不同而不同，在COPD患者与健康吸烟者大气道中，Treg细胞水平是明显升高的，而在小气道中却是显著降低的^[18, 19]^。研究^[16]^认为Treg增高表达可以介导肿瘤的免疫逃逸，促进肿瘤的进展，在肺癌中也是同样的作用。Treg细胞的增多造成免疫失衡，进而抑制Th17细胞的活性增加，削弱肺部的炎症反应，有诱发肺癌的可能，但其中的机制需进一步研究发现。近期研究^[20]^Foxp3 Tregs肿瘤基质浸润是NSCLC进展的早期事件，低淋巴细胞浸润提示不良预后，当肿瘤的少量浸润淋巴细胞高表达Foxp3 Tregs时，预后更差。肿瘤微环境中PD-L1阳性CD4^+^CD25^+^ Treg的密度可作为补充肿瘤细胞中PD-L1表达的诊断因子，并预测NSCLC对PD-1/PD-L1免疫抑制检查点治疗的反应^[21]^。

## 5 Th17细胞在COPD合并肺癌中的作用

Th17细胞是另一种T细胞亚群，IL-6和TGF-β的共同作用科促进其分化，主要分泌IL-17A、IL-17F、IL-22等细胞因子。廖晨等^[22]^研究表明Th17、IL-17A在COPD中具有重要作用，促进慢性炎症，与COPD的严重程度及吸烟有关。在COPD合并肺癌中，Th17及其相关细胞因子增加NSCLC的发生风险，并且Th17细胞对于不同分期NSCLC患者可能存在不同的影响。且国内外，动物实验及临床研究^[23, 24]^均证实，在COPD中，Th17及相关因子水平升高增加肺癌发生的风险；吸入糖皮质激素（inhaled corticosteroid, ICS）可以通过控制COPD患者的气道炎症来降低肺癌的风险，尤其在女性COPD患者中^[25]^。但另一些研究中却得出相反结论，他们发现在体外条件下，Th17细胞本身不能发挥直接杀伤肿瘤细胞的作用，而是通过刺激肿瘤细胞产生CGL2和CCL20，促进T细胞向肿瘤部位募集和启动CD8^+^ T细胞杀伤作用来实现抗肿瘤免疫。与单纯肺癌相比，Th17细胞在COPD与肺癌并存时可能存在更复杂的作用及功能。进一步深入阐明Th17细胞在COPD合并肺癌中的作用机制，基于Th17/IL-17的免疫疗法可能成为COPD合并肺癌患者治疗的新靶点。

## 6 Treg/Th17细胞在COPD与肺癌中的相互作用

Treg、Th17同属T细胞，分化微环境不同，二者在分化中相互拮抗，在作用上相互遏制。Th17细胞促进炎症反应，Treg细胞则抑制炎症反应，两者作用分歧增大、失衡，可导致机体免疫紊乱^[16]^。如前所述，在COPD中，Th17细胞表达上调而Treg细胞减少，进一步的研究^[26, 27]^表明，急性加重期和稳定期COPD患者Th17比例与Treg比例呈负相关，与IL-17水平呈正相关，提示COPD急性加重期向促炎反应转移，而稳定型COPD则转向抗炎反应。亦有研究发现，在COPD外周血中Th17及Treg细胞均高于吸烟者和健康对照组，但Th17细胞的促炎作用比Treg的抗炎功能强，Th17/Treg细胞失衡，导致持续存在的慢性气道炎症，且Th17细胞比例与肺功能呈负相关，Treg细胞增加主要与吸烟相关^[28]^。此外有研究表明，在COPD中，Th17/Treg存在动态变化，其失衡与COPD疾病程度及病程相关。多项研究^[15, 29]^发现Treg/Th17的失衡能够影响肿瘤的发生、发展：Duan等^[29]^学者发现在NSCLC患者的外周血中Th17细胞和Treg细胞均是增加的，并且伴随着的Foxp3和RORγt的上调。事实上，在促炎细胞因子和低浓度的TGF-β存在的条件下，RORγt表达进一步上调，相对的，Foxp3表达和功能受限，进而引起Th17/Treg失衡。在COPD中，各种炎性细胞因子及免疫状态可能会影响Th17/Treg平衡状态，导致炎症性免疫反应和抗炎免疫反应之间的失衡，在肺癌的发生、发展过程中起着关键的作用，但当两种疾病共存时，二者之间具体如何失衡，需要更多的研究去证实。

## 7 结语和展望

COPD与肺癌的发生、发展及预后均密切相关，戒烟仍是两者最重要的防治手段，随着两者间发病机制研究不断深入，关于COPD和肺癌的治疗药物仍在不断研究中。如今免疫治疗在医学领域尤其是恶性肿瘤中肿瘤颇有成效，COPD和肺癌的共同免疫机制是未来的研究重点。更深的理解二者的关系，从而探索出两种疾病共存时交叉治疗的可能。目前Th17/Treg在COPD合并肺癌中的规律、机制尚未明确，所以需要对它们的相互转化过程及机制进行更为深入的研究，为以后二者的治疗提供新的方向。
